# How mindfulness unlocks employee creativity: the role of workplace curiosity and performance feedback

**DOI:** 10.3389/fpsyg.2026.1789515

**Published:** 2026-04-20

**Authors:** Xia Liu, Qian Liu, Huan Liu

**Affiliations:** 1 Economics and Management School, Wuhan University, Wuhan, Hubei, China; 2 College of Health Services and Management, Xuzhou Kindergarten Teachers College, Xuzhou, Jiangsu, China; 3 School of Accounting, Shandong Technology and Business University, Yantai, Shandong, China

**Keywords:** creativity, curiosity, mindfulness, motivation, performance feedback

## Abstract

**Introduction:**

Employee traits play an important role in influencing creativity; however, it remains unclear whether mindfulness— a dynamic and adaptive personal quality— facilitates creativity. Drawing on causal orientation theory and control theory, this study aims to develop a moderated mediation model that explains how and when employee mindfulness enhances creativity.

**Methods:**

We conducted a three-wave, two-source field survey with 232 full-time employee–supervisor dyads in China. Established and validated scales were used to measure employee mindfulness, workplace curiosity, creativity, and performance feedback. We used the latent moderated structural equations (LMS) approach implemented in Mplus Version 8.3 to test the hypothesized relationships among the variables.

**Results:**

Employee mindfulness significantly boosts creativity by stimulating workplace curiosity, and this indirect relationship is moderated by performance feedback. Specifically, the positive effect of mindfulness on curiosity is stronger when employees perceive higher levels of performance feedback, which subsequently leads to greater creativity.

**Discussion:**

This study highlights the vital role of employee mindfulness in fostering creativity. By identifying curiosity as a key motivational mechanism and performance feedback as a crucial boundary condition, this work advances the literature on creativity, mindfulness, and curiosity in organizational settings.

## 1 Introduction

Creativity, a foundational source of organizational success, is defined as the production of novel and useful ideas, including the development and improvement of products and technologies, the optimization of work processes, and the creation of new business solutions (Amabile, 1988; Gong et al., 2012). Creativity often requires individuals to enter a state of flowing thinking, in which they continuously alternate between divergent and convergent thinking to solve problems or generate solutions from novel perspectives (De Dreu et al., 2008). However, the existing literature has largely conceptualized creativity as being triggered by relatively stable and static individual traits, such as proactive personality (Gong et al., 2012), procrastination (Shin and Grant, 2021), and risk-taking propensity (Xu et al., 2023). Although these studies offer rich and valuable insights, such static trait perspectives fall short of capturing the fluid and dynamic nature of creativity. Given that organizational life is increasingly filled with distractions, maintaining a high level of attentional engagement to sustain fluid, original, and flexible thinking for creative work has become ever more critical (Reina et al., 2023). In this context, a fundamental question arises: which employee traits are likely to flexibly and adaptively cope with such distractions to foster creativity?

Mindfulness refers to a receptive attention to and awareness of present-moment external (e.g., sounds) and internal (e.g., emotions) events and experiences (Brown and Ryan, 2003). It is considered a trait with dynamic self-regulation because it enables individuals to remain focused on the present moment, to broadly notice both internal and external cues, and to approach these experiences with openness and acceptance (Dane, 2011). This allows individuals to sustain high-quality aware functioning in the mind amid dynamic and unexpected conditions (Cheung et al., 2020). Accordingly, we argue that mindfulness may help employees more effectively disengage from distractions and flexibly develop their thinking and ultimately enhance creativity. Consistent with this speculation, recent studies have begun to explore the potential benefits of mindfulness for creativity-related outcomes. However, on the one hand, the majority of these studies have not fully focused on employee populations and have provided limited insight into the mechanisms through which mindfulness may influence creativity (Agnoli et al., 2018; Baas et al., 2020; Byrne and Thatchenkery, 2019; He, 2023); on the other hand, a small number of studies focusing on employees have been conducted in specific industries, yet they pay less attention to the boundary conditions under which mindfulness is effective (Cheung et al., 2020; Kivrak et al., 2025; Ngo et al., 2020). Moreover, Lebuda et al. (2016) conducted a meta-analysis and reported a positive association between mindfulness and creativity, but the relationship was relatively weak (r = 0.22). One possible explanation is that the meta-analysis included heterogeneous samples (e.g., both students and employees), which may limit the precision of our understanding of this relationship in workplace contexts. From a practice perspective, organizations have increasingly emphasized the potential value of mindfulness for creativity. Many contemporary organizations (e.g., Google, Facebook, and Microsoft) have implemented mindfulness programs to improve employees’ attentional capacity, enabling them to better integrate internal and external cues and to identify novel possibilities from fresh perspectives (Pickert, 2014; Reina et al., 2023). Taken together, the current theoretical and practical developments suggest that the relationship between mindfulness and creativity in employee populations deserves further investigation.

To further advance this insightful understanding, our goal is to examine the underlying mechanisms (i.e., why) and boundary conditions (i.e., when) through which mindfulness influences employee creativity. We draw upon causality orientations theory (COT) and control theory to propose that workplace curiosity, which is defined as a motivational state that activates individuals’ exploratory intentions and behaviors to understand the world (Chang and Shih, 2019; Loewenstein, 1994), serves as a key motivational mechanism linking mindfulness to creativity in the workplace. Research suggests that mindfulness helps employees engage in autonomous processes (Leroy et al., 2013), which are widely regarded as a vital driver of fresh and novel ideas. This indicates that employees must possess the capacity and willingness for self-directed exploration and learning when they want to create multiple ideas using divergent thinking or refine these ideas using convergent thinking (Lin et al., 2022). Accordingly, curiosity may act as a critical motivational force that bridges employee mindfulness and creativity, allowing mindful employees to fully mobilize their dynamic self-regulatory capacities to engage in broad information searching, exploration, and learning, ultimately stimulating the generation of creative ideas.

**Figure 1:**
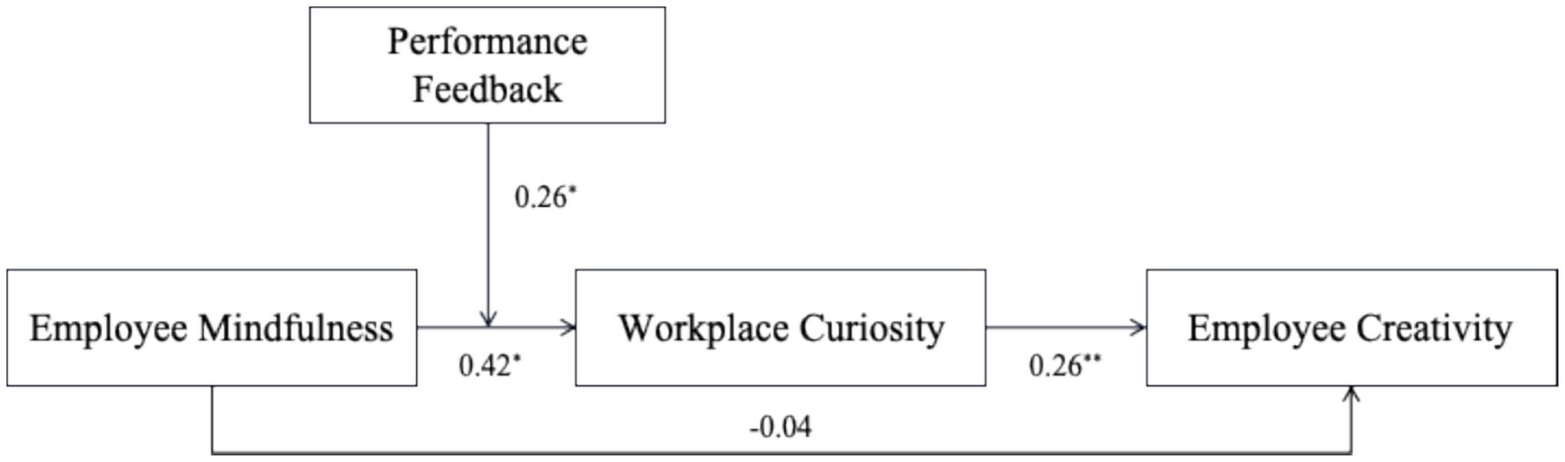
The overall research model with summarized empirical results. ^*^p < 0.05; ^**^p < 0.01.

Moreover, both COT and control theory suggest that employees who are autonomy-oriented are more likely to persist in their motivational orientation when facing a more controlling environment (Deci and Ryan, 1985; Klein, 1989). To obtain a more complete understanding of when mindfulness is more likely to foster workplace curiosity, we conceptualize performance feedback as a critical boundary condition. Although mindfulness enables employees to autonomously maintain present-moment awareness, in the absence of clear feedback about how they are performing, they may fall into the trap of being motivated but directionless (Gonzalez-Mulé et al., 2016). Performance feedback provides mindful employees with an important channel for filtering information, allowing them to more quickly identify relevant cues and enter the stages of exploration and learning, thereby enhancing their curiosity. Additionally, by offering a directional guide for employees’ present-moment attention, performance feedback further stimulates their desire to know about specific issues, prompting them to actively explore and learn to resolve knowledge gaps (Lievens et al., 2022). Overall, our conceptual model is presented in Figure 1.

The present research makes several key contributions. First, we introduce mindfulness as a dynamic antecedent factor through the lens of COT and control theory, extending our understanding of which employee traits effectively foster creativity. By showing how mindfulness unlocks employee potential through the stimulation of workplace curiosity, our study advances the understanding of the mindfulness–creativity relationship in organizational contexts. Second, we contribute to the growing body of research on workplace curiosity by deepening our understanding of its antecedents—an area that has received considerably less attention compared to the predominant focus on its outcomes—and opening new avenues for examining how curiosity is cultivated in organizations. Third, we identify performance feedback as a critical contextual factor that amplifies the extent to which mindfulness stimulates employees’ curiosity and, ultimately, their creativity. In doing so, we broaden the contextual understanding of when mindfulness is most effective in the workplace.

## 2 Theoretical background and hypotheses development

### 2.1 Theoretical framework

Causality orientations theory (COT) is a mini-theory of self-determination theory, which centers on a personality psychology framework to propose that individuals develop three causality orientations—autonomy, controlled, and impersonal orientations—that shape how they experience and interact with the world from a motivational perspective (Koestner and Levine, 2023; Deci and Ryan, 1985). Autonomy orientation reflects a tendency for behavior to be initiated and regulated by an awareness of their internal self; controlled orientation refers to the degree to which behavior is driven by external pressures, such as reward systems in the environment, or by internalized obligations that dictate how individuals believe they “should” or “must” act; impersonal orientation, represents a general tendency characterized by a lack of intentionality, initiative, and perceived control over one’s behavior (Koestner and Levine, 2023). This framework suggests that individuals (i.e., organisms) may interpret stimuli differently due to their individual differences (Deci and Ryan, 1985). Olesen et al. (2015) suggest that causality orientations can be understood as characteristic adaptations, which are shaped by both dispositional traits and contingencies in psychological contexts. Among the three orientations, the autonomy orientation particularly depends on individuals’ effective use of information to interpret and respond to environmental stimuli. Individuals with a strong autonomy orientation tend to engage in motivational processes that are aligned with their inner values and awareness, actively seeking opportunities for self-determined choice and selecting courses of action that best fit their goals and possibilities (Deci and Ryan, 1985). Therefore, COT offers an appropriate framework to help us understand how mindful employees may develop autonomy-oriented motivation (i.e., curiosity) to foster creativity.

Control theory provides a useful framework for understanding motivational processes across various fields, including personality and social psychology (Carver and Scheier, 1982; Klein, 1989). A fundamental mechanism of this theory is the feedback loop, which enables individuals to establish comparison standards and reduce discrepancies between their current state and desired goals, thereby using feedback to adjust behavior and facilitate goal attainment (Klein, 1989). Specifically, control theory suggests that individuals process information regarding their internal goals and current states, compare the two, and become aware of discrepancies between their desired and actual conditions, which subsequently triggers motivation to reduce these discrepancies (Klein, 1989). Meanwhile, COT suggests that even in controlling environments, autonomy-oriented individuals are less likely to lose their autonomous motivation and sense of self-determination (Deci and Ryan, 1985). These arguments highlight an important contextual factor for understanding how mindfulness translates into creativity through workplace curiosity—namely, supervisor’s performance feedback. Accordingly, by integrating COT and control theory, we develop a moderated mediation model to examine the full mechanism through which employee mindfulness is translated into creativity.

### 2.2 Employee mindfulness and creativity

Mindfulness involves two core characteristics: present-moment attentiveness and awareness, and an open and receptive attitude characterized by nonjudgment and nonimpulsivity (Brown and Ryan, 2003). These two aspects help individuals focus on the present moment and notice its subtle nuances, rather than becoming caught in ruminations about the past or concerns about the future (Byrne and Thatchenkery, 2019). This implies that mindfulness enables individuals to move beyond routine or formulaic thought processes, allowing employees to attend to a broader range of information and flexibly integrate it in both convergent and divergent ways (Kivrak et al., 2025). As Ryan and Deci (2017) emphasized, mindfulness facilitates greater autonomy and promotes self-congruent actions. Prior research also indicates that mindfulness is closely linked to autonomy (Brown and Ryan, 2003; Chang et al., 2015). Moreover, a recent meta-analysis found that mindfulness predicts more autonomous forms of motivation, as well as less controlled motivation and amotivation (Donald et al., 2020). In this vein, we propose that mindfulness represents an autonomy-oriented trait that shapes how individuals interpret information, thereby fostering employees’ creativity. Creativity, understood as a self-determined behavior to generate ideas that are both novel and useful (Amabile et al., 1996), inherently depends on autonomous processes, such as exploration, critical thinking, ideation, and incubation (Lin et al., 2022). Accordingly, the ability for employees to maintain broad awareness and capture diverse information is essential for facilitating creative activities (Cheung et al., 2020; Lebuda et al., 2016).

When employees are mindful, they are better able to monitor distracting thoughts and disengage their attention from the distractor, reduce anxiety and stress, and gain greater clarity about their own thoughts, rather than expend time and energy struggling with reality (Baas et al., 2020). As a result, mindfulness enhances task concentration (Shapiro et al., 2006), facilitates information gathering and incubation processes (Cheung et al., 2020), and enhances insight and idea fluency (Ostafin and Kassman, 2012). Moreover, because mindfulness reduces individuals’ tendency to rely on habitual responses, it enables employees to engage in higher-quality thinking when searching for solutions (Ngo et al., 2020). Current literature also highlights that mindfulness can enhance creative outcomes (Agnoli et al., 2018; Baas et al., 2020; Byrne and Thatchenkery, 2019; He, 2023; Kivrak et al., 2025; Lebuda et al., 2016; Ngo et al., 2020). For example, Byrne and Thatchenkery (2019) found that employees’ mindfulness training positively impacted creativity in the moment and over time. Kivrak et al. (2025) identified a similar pattern in the media industry. In addition, Lebuda et al. (2016) conducted a meta-analysis to find that mindfulness is relatively related to creativity. Although this meta-analysis was not exclusively focused on employee samples, the accumulated evidence provides a strong basis for predicting the effect of employees’ mindfulness on creativity. Therefore, we propose the following hypothesis,

*H1:* Employee mindfulness is positively related to creativity.

### 2.3 The mediating role of workplace curiosity

Workplace curiosity is rooted in human motivation and drives employees to actively search for new knowledge and things, linking novelty and challenge with opportunities for growth (Chang and Shih, 2019). As such, curiosity reflects individuals’ desire to acquire new knowledge, experiences, and information, as well as to explore the unknown. Recent work has acknowledged curiosity is a clear foundation for a more creative life in modern organizations (Fredericks, 2022; Koutstaal et al., 2022; Schutte and Malouff, 2020). When individuals are not constrained by their habitual ways of thinking and are able to view the world from multiple perspectives, curiosity enables them to approach the unknown with an open mind. When this process is self-initiated and autonomous, individuals will conduct self-congruent action to expand their knowledge, thinking, and skills, which in turn enhances creativity (Ryan and Deci, 2017; Schutte and Malouff, 2020). Based on COT, we propose that workplace curiosity is a vital mechanism representing autonomy-oriented motivation through which employees’ mindfulness translates into creativity.

First, COT suggests that individuals with an autonomy-oriented trait tend to interpret both internal and external cues as autonomy-supportive or informational, which enables them to make flexible choices. Mindfulness closely reflects this tendency, as it involves an active awareness of the present moment that allows employees to direct their attention to information unfolding in both internal and external environments (Brown and Ryan, 2003; Dane, 2011). Such alive awareness allows employees to be less preoccupied with past ruminations or future fantasies (Bishop et al., 2004; Reina et al., 2023), enabling them to focus on their immediate experiences and access a broader range of information (Langer, 1989), thereby strengthening their connection with the surrounding environment (Arendt et al., 2019). These informational connections further guide employees’ choices to act with greater freedom and engage in an autonomy motivational process (Deci and Ryan, 1985) that aligns with their inner awareness and thoughts, and ultimately activates their exploratory intentions in their work area (Chang and Shih, 2019; Loewenstein, 1994).

Second, mindfulness enables employees to capture fresh experiences and information, fostering a heightened interest in future exploration and reflection (Lievens et al., 2022). The activation of curiosity often involves suspending heuristics and past experiences, which is a critical precondition for its emergence, as it helps employees resist self-doubt and self-critical thoughts (Lievens et al., 2022). When being mindful, employees adopt an open and unbiased attitude toward information processing (Bishop et al., 2004; Shapiro et al., 2006), immersing themselves in an expansive informational environment characterized by novel cognitive experiences (Langer, 1989), rather than being anchored in established knowledge structures. That said, by adopting a nonjudgmental stance and decoupling their thoughts from their emotions, employees are able to navigate diverse information and perspectives with greater objectivity, which improves the quality of the information processing in the mind (Cheung et al., 2020). Drawing on COT, mindful employees are more likely to autonomously detect inconsistencies or gaps in their existing knowledge frameworks and, in turn, significantly amplify their desire to know—that is, it evokes a strong autonomy motivation to think, learn, and explore in their work area, namely, fosters workplace curiosity (Lievens et al., 2022).

Previous research has shown that mindfulness can help employees enhance autonomous motivation (Marion-Jetten et al., 2022) and improve motivational control (Dust et al., 2022), which strengthens our inference. Together, we suggest that mindfulness is positively associated with curiosity by enabling employees to accumulate a large amount of information, identify knowledge gaps, and stimulate their intrinsic interest in exploration. We hence propose the following hypothesis,

*H2:* Employee mindfulness is positively related to workplace curiosity.

Next, we suggest that enhanced curiosity will further boost employee creativity. Creativity often requires employees to develop unique ideas that differ from traditional solutions in a flexible and effective manner (Zhou and Hoever, 2014), which requires a large amount of divergent and convergent thinking. Thus, the emergence of creativity typically relies on an autonomous, exploratory, and adventurous mindset, which curiosity is uniquely positioned to provide (Chang and Shih, 2019). First, employees with higher levels of curiosity actively engage in broad exploration and learning driven by intrinsic interests (Lievens et al., 2022). Curiosity essentially reflects the desire to explore and learn about novel and uncertain phenomena to solve problems (Lievens et al., 2022). In the workplace, when faced with diverse information flows as well as uncertain or complex situations, curious employees are excited and interested to actively seek sensory stimuli, engage in cognitive observation, or raise questions to acquire new information, thereby satisfying their need for acquiring a broader scope of heterogeneous knowledge (Chang and Shih, 2019; Hardy et al., 2017). COT suggests that such autonomous exploration helps them continuously search for, integrate, and converge valuable information and knowledge, as well as generate multiple fresh ideas, thereby creating useful and unconventional solutions.

Second, employees with higher curiosity are better at identifying unique information that differs from what is familiar, which helps them bridge existing knowledge gaps and alleviate the discomfort arising from such gaps (Lievens et al., 2022). Extensive exploration and continuous learning allow these employees to accumulate knowledge while constantly encountering novel information. However, such information—being different from existing knowledge—can trigger significant cognitive discomfort, motivating them strongly to close these knowledge gaps (Lievens et al., 2022). Therefore, curiosity drives them to persistently explore, learn, and reflect, generating new and useful ideas to resolve these discrepancies. Existing research suggests that curious employees may excel at idea generation because they are willing to embrace risk, venture into new cognitive paths, and experiment with novel ideas (Chang and Shih, 2019). Thus, we suggest that curious employees are able to fully leverage their capacity for autonomous exploration and learning, and ultimately translate their curiosity into higher levels of creativity. We thus predict that,

*H3:* Workplace curiosity is positively associated with employee creativity.

Integrating Hypotheses 1–3, we propose that workplace curiosity serves as a key motivational mechanism through which employee mindfulness is translated into creativity. Although prior research has examined the link between mindfulness and creativity through mechanisms such as intrinsic motivation, cognitive flexibility, and creative process engagement (Cheung et al., 2020; Kivrak et al., 2025), these explanations may not fully capture the underlying process. From the perspective of COT, workplace curiosity may provide a novel motivational lens for understanding this relationship. Specifically, we argue that employee mindfulness reflects an autonomy-oriented trait that allows individuals to attend to present-moment experiences with reduced automaticity and greater openness to internal and external stimuli. Such awareness enables employees to adopt multiple perspectives and engage in self-initiated exploration, thereby fostering an autonomy-oriented motivational state—workplace curiosity. In turn, heightened curiosity facilitates the encoding, integration, and recombination of knowledge, ultimately promoting the generation of novel and useful ideas, that is, employee creativity. Overall, we propose that,

*H4:* Workplace curiosity positively mediates the relationship between employee mindfulness and creativity.

### 2.4 The moderating role of performance feedback

Performance feedback refers to information about the actual performance or actions of a system used to control the future actions of a system (Gonzalez-Mulé et al., 2016). According to control theory (Carver and Scheier, 1982; Klein, 1989), supervisor performance feedback provides employees with information about discrepancies between their current performance and established standards (Gonzalez-Mulé et al., 2016), which in turn, motivates them to monitor and adjust their goals to better align with these standards. More importantly, from a control theory perspective, employees with higher self-awareness are more likely to recognize the gap between their actual and desired performance when receiving feedback, which in turn strengthens their motivation to reduce such discrepancies (Klein, 1989). At the same time, COT suggests that in controlling environments, autonomy-oriented individuals are more likely to maintain their autonomy-related motivation and engage in self-determined behaviors (Deci and Ryan, 1985). By integrating the core arguments of control theory and COT, we propose that performance feedback serves as a critical boundary condition that strengthens the positive relationship between mindfulness and workplace curiosity.

When employees perceive a high level of performance feedback, they receive clear signals about which goals and tasks are most important, as well as where their current performance falls short (Gonzalez-Mulé et al., 2016). More importantly, supervisors often help employees adjust their action direction and goals based on the specific issues they encounter, thereby encouraging autonomous learning (Lee et al., 2023). Thus, while mindfulness broadens employees’ attentional breadth and enhances their intention in exploring and learning new things, performance feedback reinforces their sense of goals and awareness of performance discrepancies during information processing. Based on COT and control theory, this information can help heighten employees’ goal clarity and discrepancy awareness, amplifying employees’ desire to know and strengthening their motivation to close these gaps (Lievens et al., 2022). Therefore, we argue that under conditions of a high level of performance feedback, the positive effect of mindfulness on curiosity will be strengthened.

Conversely, when employees receive a low level of performance feedback, they struggle to clarify the work goals they are expected to achieve and to identify problems in their current performance. In other words, low levels of performance feedback make employees’ goals ambiguous and obscure their understanding of what the organization expects from them (Gonzalez-Mulé et al., 2016). Although performance feedback is generally considered an important cue that stimulates exploration and learning, employees who cannot obtain clear evaluations from their supervisors have little insight into what they are doing well and which areas require enhancement (Chambers et al., 2024). Under such circumstances, although mindfulness can help employees expand their attentional breadth and accumulate extensive information and knowledge, the absence of clear feedback prevents them from recognizing the discrepancies between this new information and their current performance level. As a result, they are less likely to be motivated to engage in problem-focused information search and exploratory learning (Lievens et al., 2022). Therefore, we argue that low levels of performance feedback weaken the positive effect of mindfulness on curiosity. Thus, we predict that,

*H5:* Performance feedback moderates the positive relationship between employee mindfulness and curiosity, such that the relationship is more positive at higher (versus lower) levels of performance feedback.

Integrating Hypotheses 1–5 and drawing on control theory and COT, we propose that performance feedback functions as an important contextual control factor that helps mindful employees attend to and interpret performance-related information from their supervisors. This awareness increases the likelihood that employees recognize discrepancies between their ideal and current performance. As a result, they may experience stronger curiosity, motivating them to engage in autonomous exploration, learning, and knowledge integration, which in turn facilitates creativity (Chang and Shih, 2019; Lievens et al., 2022). Accordingly, we propose that the indirect effect of mindfulness on creativity via curiosity will be strengthened when performance feedback is high, whereas this indirect relationship will be weakened when performance feedback is low. Stated formally,

*H6*: Performance feedback moderates the indirect association between employee mindfulness and creativity via curiosity, such that the indirect association is more positive at higher (versus lower) levels of performance feedback.

## 3 Methods

### 3.1 Sample and procedures

Data was collected from full-time employees across several industries in China, including finance, service, production and operation, marketing, and technology research and development. Eligible employees were required to hold positions that involved updating work approaches, implementing changes, and generating new ideas to adapt to a dynamic business environment. We contacted full-time managers working in these industries and asked them to assist in recruiting eligible employees and their immediate supervisors to participate in our survey. We then created online questionnaire links through Wenjuanxing.com, a widely used data collection platform in China. The managers distributed the corresponding survey links to employees and supervisors who voluntarily agreed to participate. Before distributing the survey, managers generated unique, non-identifiable codes for each participating employee and their immediate supervisor to facilitate the matching of responses. This procedure helped ensure that participants could respond with confidence, without concern that their answers would be known to their subordinates, colleagues, or supervisors. In addition, participants were informed that their participation was voluntary, anonymous, and confidential, and that the data would be used solely for academic research purposes.

To mitigate potential common method bias, data were collected across three time points, each separated by a two-week interval. At Time 1, employees reported their demographic characteristics, mindfulness, and performance feedback, as well as a series of controls (i.e., positive and negative affect, intrinsic motivation, and need for cognition). At Time 2, employees rated workplace curiosity. At Time 3, their immediate supervisor rated the employee’s creativity. From the initial 321 employees, 266 completed both the Time 1 and Time 2 surveys, the total response rate was 82.87%. In the last phase, we received 232 responses from their immediate supervisors, the response rate was 87.22%. Our final sample included 43% male and 57% female respondents. Employees’ average age was 32.78 (SD = 7.88), and average organizational tenure was 3.63 (SD = 3.61). 88.36% of them had college or above education.

### 3.2 Measures

Owing to our samples being from China, to ensure the measurement equivalence to the Western scale, we followed the commonly used back-translation procedure to administer the survey in Chinese (Brislin, 1980).

#### 3.2.1 Employee mindfulness

Employees were invited to report the extent to which they experienced mindfulness at work using the 22-item mindfulness at work scale by Hülsheger and Alberts (2021). The measure used a six-point Likert scale (1 *= strongly disagree,* 6 *= strongly agree*). Sample items are “When I experience unpleasant emotions during work, they easily take over (r)”, “At work, I criticize myself for having irrational or inappropriate emotions (r)”, and “During work, I find it easy to stay focused on the task at hand”. The reliability coefficient was 0.89.

#### 3.2.2 Workplace curiosity

Participants indicated the extent to which they agree with workplace curiosity in the last two weeks on a 6-item scale ranging from 1 *= strongly disagree* to 7 *= strongly agree* (Tolentino et al., 2014). Sample items are “exploring my surroundings*”* and *“*looking for opportunities to grow as a person”. The reliability coefficient was 0.97.

#### 3.2.3 Performance feedback

Employees reported their perception of performance feedback from their immediate supervisors with the 3-item scale from Gonzalez-Mulé et al. (2016), ranging from 1 *= strongly disagree* to 7 *= strongly agree*. A sample item is “I seldom receive feedback from my supervisor on the quality of work I have done (reverse)”. The reliability coefficient was 0.92.

#### 3.2.4 Employee creativity

Immediate supervisors were invited to report the employee’s creativity with the 4-item scale from Farmer et al. (2003), ranging from 1 *= strongly disagree* to 7 *= strongly agree*. A sample item is “This employee tries new ideas or methods first”. The reliability coefficient was 0.94.

#### 3.2.5 Control variables

To avoid possible alternative explanations, we controlled some important variables in our analyses. First, we controlled for positive and negative effects since they can influence mindful employees’ work outcomes (Malinowski et al., 2015). We measured employees’ positive and negative effects with the 10-item I-PANAS-SF scales from Karim et al. (2011), which included five positive affect items (i.e., active, determined, attentive, inspired, and alert; *α* = 0.91), and five negative affect items (i.e., afraid, nervous, upset, hostile, and ashamed; α = 0.91). The response scales ranged from 1 *= never* to 6 *= always*. Second, we controlled for two constructs (i.e., need for cognition and intrinsic motivation) that were more closely associated with curiosity (for a review, see Lievens et al., 2022) and creativity (Amabile, 1988; Wu et al., 2014). We used 8 items of the need for cognition scale from Cacioppo et al. (1984) to apply in a work context. The response scale ranged from 1 *= strongly disagree* to 5 *= strongly agree*. Sample items were “I like to have the responsibility of handling a situation that requires a lot of thinking” and “I really enjoy a task that involves coming up with new solutions to problems”. The reliability coefficient was 0.90. Intrinsic motivation was assessed with a 4-item scale from Grant (2008), which ranged from 1 *= strongly disagree* to 5 *= strongly agree*. A sample item was “I enjoy the work itself”. The reliability coefficient was 0.93. Finally, we controlled for the employees’ gender (0 = *Male*, 1 = *Female*), age (in *years*), education level (1 = *High school or equivalent*; 2 = *Some college*; 3 = *College graduate* (4 years); 4 = *Master’s degree or higher*), and organizational tenure (in *years*) to rule out the demographic difference. There are no results that changed if we excluded these control variables from our analyses.

## 4 Results

### 4.1 Measurement model

We conducted several analyses to test whether the core variables were distinct. First, we performed a series of confirmatory factor analyses (CFAs) with Mplus Version 8.3 to verify the discriminant validity of the theoretical model. We created four parcels for employee mindfulness based on its four dimensions according to Williams and O’Boyle (2008). All other scales were indicated by specific items. As shown in Table 1, the results showed that the four-factor model was a better fit for the data (χ^2^_[113]_ = 173.02, TLI = 0.98, CFI = 0.98, RMSEA = 0.05, SRMR = 0.04) than any other models (316.85 ≤ Δχ^2^s ≤ 1732.78, 3 ≤ Δdfs ≤ 6, *p*s < 0.001). Then, we calculated the average variance extracted (AVE) and composite reliability (CR) to examine the content and structure validity. The AVE and CR scores for employee mindfulness were 0.60 and 0.85, for workplace curiosity were 0.83 and 0.97, for leader performance feedback were 0.80 and 0.92, and for employee creativity were 0.80 and 0.94. For all focal constructs, the AVE and CR scores were greater than the cutoff values of 0.5 and 0.6 (Fornell and Larcker, 1981), revealing that each construct has greater internal consistency and convergent validity. Moreover, the square roots of all AVEs were greater than the correlations (Fornell and Larcker, 1981), indicating acceptable discriminant validity. Lastly, we followed Podsakoff et al.’s (2003) suggestions to conduct Harman’s single-factor test on the potential threat of common method bias. Results showed that the single unrotated factor accounts for 28.91% of the total variance, lower than 40%. Thus, the common method bias is not a serious concern in our study.

**Table 1:** Confirmatory factor analysis results of construct discriminant validity.

Model	Δχ^2^(Δ*df*)	χ^2^(*df*)	RMSEA	CFI	TLI	SRMR
Four-factor model: EM, WC, PF, EC		173.02(113)	0.05	0.98	0.98	0.04
Three-factor model: EM + PF, WC, EC	316.85(3)**	489.87(116)	0.12	0.90	0.88	0.11
Three-factor model: EM + WC, PF, EC	405.69(3)**	578.71(116)	0.13	0.87	0.85	0.13
Three-factor model: EM, PF, WC + EC	810.04(3)**	983.06(116)	0.18	0.76	0.72	0.15
Two-factor model: EM + PF, WC + EC	1124.79(5)**	1297.81(118)	0.21	0.68	0.63	0.18
Two-factor model: EM + PF + WC, EC	924.01(5)**	1097.03(118)	0.19	0.73	0.69	0.17
Single-factor model: EM + PF + WC + EC	1732.78(6)**	1905.80(119)	0.25	0.51	0.44	0.22

*N* = 232. EM = Employee mindfulness. WC = Workplace curiosity. PF = Performance feedback. EC = Employee creativity. χ^2^ = chi-square value given by Maximum Likelihood estimator. Δχ^2^ = difference chi-square. df = degrees of freedom. Δdf = difference in df. RMSEA = root mean squared error of approximation. CFI = comparative fit index. TLI = Tucker-Lewis index. SRMR = standard root mean square residual. ** *p* < 0.01 (two-tailed).

### 4.2 Hypothesis testing

Table 2 presented the means, standard deviations, and correlations of our focal variables. As expected, employee mindfulness showed a positive correlation with workplace curiosity (*r* = 0.38; *p* < 0.01), and workplace curiosity was positively associated with employee creativity (*r* = 0.23; *p* < 0.01). These results provided preliminary evidence for our hypothesis testing.

**Table 2:** Means, standard deviations, and correlations among the study variables.

Variable	Mean	SD	1	2	3	4	5	6	7	8	9	10	11
1. Gender	0.57	0.50											
2. Age	32.78	7.88	−0.05										
3. Education	2.63	0.94	0.06	−0.35**									
4. Organizational tenure	3.63	3.61	0.08	0.47**	0.11								
5. Positive affect	3.59	0.64	0.06	0.13*	−0.06	−0.10							
6. Negative affect	2.28	0.71	0.01	0.05	−0.14*	−0.05	−0.30**						
7. Need for cognition	3.50	0.53	−0.09	0.03	−0.06	−0.13	0.51**	−0.19**					
8. Intrinsic motivation	3.61	0.68	0.07	0.13	−0.13*	−0.07	0.59**	−0.23**	0.58**				
9. Employee mindfulness	4.20	0.55	0.10	0.08	0.07	0.02	0.56**	−0.55**	0.42**	0.42**			
10. Performance feedback	5.06	1.32	0.06	−0.06	0.16*	0.01	0.25**	−0.26**	0.09	0.19**	0.48**		
11. Workplace curiosity	5.36	0.92	0.06	0.03	−0.04	−0.07	0.40**	−0.15*	0.35**	0.35**	0.38**	0.14*	
12. Employee creativity	5.31	1.01	0.01	0.07	−0.06	−0.14*	0.14*	0.18**	0.10	0.03	0.01	0.01	0.23**

*N* = 232. **p < 0.01; *p < 0.05 (two-tailed).

We used Mplus Version 8.3 to conduct latent moderate structural equations (LMS) to test our full model with bias-corrected CIs based on 1,000 random samples for the indirect effects and conditional indirect effects (Cheung and Lau, 2017). Our path analyses were reported in Table 3 and the summarized path coefficient was presented in Figure 1. H1 predicted that employee mindfulness would be positively related to employee creativity. However, the results indicate that the direct effect of mindfulness on creativity is not significant (*Effect* = −0.04, *S.E.* = 0.17, 95% CI = [−0.37, 0.31]); therefore, H1 is not supported. Supporting H2 and H3, employee mindfulness was positively related to workplace curiosity (*Effect* = 0.42, *S.E.* = 0.17, 95% CI = [0.10, 0.75]), and workplace curiosity was positively associated with employee creativity (*Effect* = 0.26, *S.E.* = 0.08, 95% CI = [0.10, 0.41]). Further, performance feedback interacted with employee mindfulness on curiosity was significant (*Effect* = 0.26, *S.E.* = 0.11, 95% CI = [0.04, 0.46]). We then test the simple slopes at high (+1 SD) and low (−1 SD) levels of performance feedback, the results indicated that the relationship between employee mindfulness and workplace curiosity was significant when performance feedback was high (*Effect* = 0.73, *S.E.* = 0.21, 95% CI = [0.30, 1.14]), but nonsignificant when performance feedback was low (*Effect* = 0.10, *S.E.* = 0.22, 95% CI = [−0.29, 0.54]), supporting H5.

**Table 3:** Estimated effects and bias-corrected confidence intervals.

Path	Effect	S.E.	95% BCLL	95% BCUL
Employee mindfulness → workplace curiosity	0.42	0.17	0.10	0.75
Performance feedback → workplace curiosity	0.00	0.07	−0.13	0.13
Employee mindfulness × performance feedback → workplace curiosity	0.26	0.11	0.04	0.46
Employee mindfulness → employee creativity	−0.04	0.17	−0.37	0.31
workplace curiosity → employee creativity	0.26	0.08	0.10	0.41
Index	0.07	0.04	0.004	0.147

*N* = 232; S.E. = estimated standard error; *p*-value = *p* value under the assumption of normal distribution; BCLL = lower limit of bias-corrected bootstrap confidence interval; BCUL = upper limit of bias-corrected bootstrap confidence interval. Results are reported from models including control variables. For brevity, the coefficients of control variables are not presented.

We then test the indirect effect and the conditional indirect effects. The results showed employee mindfulness was positively associated with employee creativity through workplace curiosity (*Indirect effect* = 0.11, *S.E.* = 0.05, 95% CI = [0.02, 0.22]), supporting H4. Moreover, results showed that the interaction term for employee mindfulness and performance feedback was significantly related to employee creativity via workplace curiosity (*Index: Effect* = 0.07, *S.E.* = 0.04, 95% CI = [0.004, 0.147]). Employee mindfulness was positively associated with employee creativity through curiosity at higher levels of performance feedback (*Effect* = 0.19, *S.E.* = 0.08, 95% CI = [0.05, 0.36]), but not at lower levels of performance feedback (*Effect* = 0.03, *S.E.* = 0.06, 95% CI = [−0.09, 0.14]), thus supporting H6 (see Table 4).

**Table 4:** Moderated mediation effect of mindfulness on creativity at various values of performance feedback.

Path	Effect	S.E.	95% BCLL	95% BCUL
+1 sd	0.19	0.08	0.05	0.36
Mean	0.11	0.05	0.02	0.22
−1 sd	0.03	0.06	−0.09	0.14

*N* = 232; +1 sd = one standard deviation above mean; −1 sd = one standard deviation below mean; BCLL = lower limit of bias-corrected bootstrap confidence interval; BCUL = upper limit of bias-corrected bootstrap confidence interval.

In addition, most control variables did not show significant effects on workplace curiosity or creativity. One noteworthy finding is that negative affect was positively related to creativity (*Effect* = 0.30, *S.E.* = 0.11, 95% CI = [0.07, 0.52]). Although this result may appear somewhat unusual, it is consistent with the context of the present study and prior research. First, previous studies suggest that negative affect can serve as an important driver of creativity (De Dreu et al., 2008; George and Zhou, 2002, 2007; Bledow et al., 2013). Negative affect often signals that something is problematic, prompting individuals to identify and resolve issues more systematically. As a result, it encourages a more bottom-up, detail-oriented, and analytical approach to processing information, which can facilitate creative problem solving (George and Zhou, 2007). Second, prior research indicates that negative affect can be functional because it alerts individuals to potential shortcomings, directs attention to the present situation rather than prior assumptions, and motivates greater effort to improve the situation (George and Zhou, 2002). This mechanism aligns with the role of mindfulness in our model, which emphasizes attending to present experiences rather than relying on preexisting assumptions, and may therefore amplify the functional effects of negative affect. Third, according to Litman (2008), curiosity can be divided into interest-type (I-type) curiosity and deprivation-type (D-type) curiosity. I-type curiosity reflects the desire to experience the positive feelings associated with learning (“I would love to know”), whereas D-type curiosity reflects the motivation to reduce the discomfort caused by a lack of information (“I need to know”). However, in the present study, workplace curiosity was not differentiated into these two types. Therefore, it is possible that the presence of D-type curiosity made the positive effect of negative affect on creativity more salient in our findings. Taken together, this finding provides additional insight into the role of negative affect in creativity and offers a valuable direction for future research to explore both positive and negative pathways associated with mindfulness.

### 4.3 Additional analysis

Although we controlled for intrinsic motivation—an important motivational mechanism underlying creativity (e.g., Amabile, 1988; Kivrak et al., 2025)—we further incorporated intrinsic motivation into the structural equation model to examine competing models, thereby clarifying the unique mediating role of curiosity in the present model and ruling out alternative explanations. Specifically, we constructed three competing models: (a) a dual-mediator model in which workplace curiosity and intrinsic motivation were specified as parallel mediators; (b) a sequential mediation model (mindfulness → curiosity → intrinsic motivation → creativity); and (c) an alternative sequential mediation model (mindfulness → intrinsic motivation → curiosity → creativity). We compared the mediation path coefficients and model fit indices of the hypothesized model with those of the three competing models, including the Akaike Information Criterion (AIC) and Bayesian Information Criterion (BIC), where smaller values indicate better model fit (Kuha, 2004). The results showed that the mediation effects in all three competing models were not significant (Model a: *Effect* = −0.04, *S.E.* = 0.03, 95% CI = [−0.11, 0.02]; Model b: *Effect* = −0.00, *S.E.* = 0.00, 95% CI = [−0.008, 0.004]; Model c: *Effect* = 0.00, *S.E.* = 0.01, 95% CI = [−0.01, 0.01]). In addition, the AIC and BIC values of these competing models were all higher than that of the hypothesized model (hypothesized model: AIC = 7475.07, BIC = 7726.68; Model a: AIC = 8723.58, BIC = 9047.57; Model b: AIC = 8723.53, BIC = 9047.52; Model c: AIC = 8723.64, BIC = 9047.64). Taken together, these findings provide additional support for our proposed model and suggest that intrinsic motivation is unlikely to account for the additional mechanism in this study.

## 5 Conclusions and discussion

### 5.1 Conclusion

Drawing on COT and control theory, this study explores the mechanism and under what conditions employee mindfulness enhances creativity. Based on a multi-source, multi-wave empirical study of 232 supervisor–employee dyads in China, the main findings are as follows:

First, our results show that mindfulness does not have a significant direct effect on creativity (H1 was not supported). Instead, mindfulness promotes creativity indirectly through the activation of workplace curiosity as a motivational state (H2–H4 were supported). This finding suggests that the influence of mindfulness on creativity is more complex than a simple direct relationship. In our study, workplace curiosity captures how mindfulness facilitates autonomy-oriented motivation, which in turn fosters creative outcomes. In fact, prior research has reported mixed findings regarding the motivational role of mindfulness, which may help explain why its direct effect on creativity was not significant in our study. On the one hand, some scholars highlight the motivationally enhancing role of mindfulness. For example, Kivrak et al. (2025) found that mindfulness can increase intrinsic motivation, and Dust et al. (2022) demonstrated that mindful employees are better able to continuously adjust goal priorities and the direction of their efforts, thereby strengthening their motivational control. On the other hand, more critical perspectives suggest that mindfulness may increase individuals’ focus on the present task while reducing attention to future goals, which could potentially weaken task motivation (Hafenbrack and Vohs, 2018). These mixed findings further underscore the complexity of mindfulness in activating motivational states. Nevertheless, by highlighting workplace curiosity as an autonomy-oriented motivational state that mediates the relationship between mindfulness and creativity, our study provides additional evidence for the motivational benefits of mindfulness and enriches the understanding of its underlying motivational mechanisms.

Second, a key boundary condition for the effective operation of the above mechanism is performance feedback (H5 and H6 were supported). As a fundamental feature of job design, performance feedback provides important guidance on how employees should engage in their work activities. Leaders, as the primary source of performance feedback, typically evaluate employees’ work performance on a regular basis and provide feedback to ensure that their behaviors align with organizational expectations. Accordingly, this job characteristic helps employees clarify what is important and how to engage in their work more effectively, making it particularly conducive to strengthening the curiosity of mindful employees. Under high levels of performance feedback, the information conveying clear goals and explicit directions for improvement is incorporated into mindful employees’ minds, enabling them to better engage in autonomous processes. This facilitates their efforts to seek more useful information, explore new directions, and acquire broader knowledge. In doing so, it enhances their desire to know and drives them to narrow the gap between their current and ideal behavior (Lievens et al., 2022), thereby amplifying the positive effect of mindfulness on curiosity. Consequently, this finding provides timely and deeper insight into how mindfulness influences curiosity and its subsequent impact on creativity.

### 5.2 Theoretical contribution

This study makes several key contributions. First, we introduce mindfulness as an important dispositional antecedent, thereby enriching our understanding of how employee creativity is driven in the workplace. Prior creativity research has primarily focused on relatively static personality traits—such as proactive personality (Gong et al., 2012), procrastination (Shin and Grant, 2021), and risk-taking propensity (Xu et al., 2023)—as key drivers of creativity. However, given that creativity often requires fluent information processing, the generation of unusual ideas and insights, and flexible cognitive perspectives, personal traits characterized by dynamic and attentive observation may play a particularly important role in facilitating divergent thinking and creative idea generation. By introducing mindfulness as a highly adaptive and dynamic individual trait, we found that mindfulness enables employees to more effectively observe and capture diverse internal and external information, which stimulates workplace curiosity, promotes broader exploration and learning, and ultimately enhances creativity. As a result, doing so adds a novel trait-based antecedent that advances a deeper understanding of how employee creativity can be fostered.

Second, by unpacking the creative consequences of mindfulness and elucidating the processes through which these effects emerge, this study extends the literature on mindfulness in organizational management. Previous research on mindfulness has largely focused on its effects on well-being. For example, employee mindfulness has been found to reduce emotional exhaustion (Hülsheger et al., 2013; Schultz et al., 2015), alleviate stress (Johnson et al., 2021), and improve sleep quality (Hülsheger et al., 2014). As mindfulness has increasingly been introduced into workplace contexts, scholars have also begun to examine its influence on work-related outcomes. For instance, prior studies suggest that mindfulness can enhance job satisfaction and work engagement (Hülsheger et al., 2013; Leroy et al., 2013). Although some research has begun to explore the relationship between mindfulness and creativity, the existing evidence remains limited. For example, Lebuda et al. (2016) conducted a meta-analysis and found that mindfulness is positively related to creativity. However, their study did not fully focus on a broad employee population. To date, only a small number of studies have examined this relationship within specific contexts. For instance, Kivrak et al. (2025), in the media industry, found that mindfulness can promote creativity by enhancing intrinsic motivation and cognitive flexibility. Therefore, whether mindfulness can generally promote creativity across broader organizational settings has not yet been thoroughly examined. To further advance this insight, we emphasize that mindfulness fosters employees’ workplace curiosity, which enables them to engage in the intentional search for, exploration of, learning from, and encoding of diverse information (Cheung et al., 2020), ultimately enhancing creativity. This finding broadens the understanding of the relationship between mindfulness and creativity in the field of organizational management.

Moreover, drawing on COT and control theory, we identify workplace curiosity as a critical motivational mechanism through which mindfulness enhances creativity, thereby offering a valuable extension to the literature on the motivational mechanisms of mindfulness. Prior research on mindfulness and motivation has yielded mixed findings: while some scholars argue that mindfulness enhances motivational control, intrinsic motivation, and autonomous motivation (e.g., Dust et al., 2022; Kivrak et al., 2025; Marion-Jetten et al., 2022), others contend that mindfulness may undermine task motivation (e.g., Hafenbrack and Vohs, 2018). Our findings lend support to the former perspective by demonstrating that mindfulness facilitates autonomous processes that stimulate workplace curiosity, which in turn fosters creativity. In doing so, our study enriches current understanding of the motivational mechanisms underlying mindfulness.

Third, this study contributes to the growing literature on workplace curiosity by advancing our understanding of its antecedents. Although scholars widely acknowledge that curiosity leads to a range of beneficial outcomes—such as increased work engagement (Schweitzer et al., 2023), job performance (Lievens et al., 2022), and creativity (Chang and Shih, 2019)—empirical exploration of its antecedents remains scarce and largely conceptual (Hinrichs et al., 2023). Consequently, how organizations can effectively cultivate employees’ curiosity to maintain performance advantages is still not well understood. To advance novel insights on this research line, drawing on the COT and control theory, we identify that mindfulness is an important antecedent of workplace curiosity. Our findings not only expand the knowledge of what individual characteristics trigger curiosity but also open up new avenues for future research on the processes through which curiosity is fostered in the workplace.

Fourth, we identify a critical contextual factor (i.e., performance feedback) that strengthens the extent to which mindfulness stimulates employees’ curiosity and ultimately enhances their creativity. Prior research has frequently examined performance feedback alongside other job characteristics when predicting employees’ job satisfaction and work engagement (Jong, 2016; Lechermeier et al., 2020). However, relatively little attention has been paid to how performance feedback, as a key job characteristic, interacts with individual traits to shape more distinctive employee outcomes. Our study helps address this limitation. Our findings show that when employees receive higher levels of performance feedback, they become more motivated to autonomously channel their curiosity toward creative outcomes. This is because performance feedback enables mindful employees to better understand their shortcomings and future directions for exploration, thereby enhancing their sense of autonomy and energizing their exploratory and learning orientation. This discovery also enriches the boundary conditions under which mindfulness facilitates autonomous processes and broadens the contextual understanding of its effectiveness in the workplace (Good et al., 2016).

### 5.3 Managerial implications

Our study also offers important implications for organizational management. First, our findings suggest that mindfulness can promote employee creativity, particularly in the context of supervisor performance feedback. Accordingly, organizations may not only promote mindfulness training among employees (Reina et al., 2023) but also encourage leaders to provide more informational rather than controlling performance feedback to mindful employees. Such practices may amplify the benefits of mindfulness because, when provided with sufficient external information, employees who attend to the present moment with openness and nonjudgment are more likely to broaden their motivation to seek information, explore new ideas, and engage in learning while reducing tunnel vision, which in turn stimulates workplace curiosity and ultimately enhances creativity.

Second, our finding that workplace curiosity represents a key motivational mechanism through which mindfulness influences creativity suggests that organizations should intentionally emphasize the cultivation of curiosity in employee training and development. Our study indicates that curiosity is not a fixed personality trait but can be stimulated and shaped through appropriate managerial practices (Lievens et al., 2022). According to our findings, organizations can encourage employees to mindfully adopt a more open orientation toward diverse sources of information, providing multiple tools and channels for information search and learning, and offering regular, constructive feedback on their performance. Together, these approaches may have the potential to more effectively stimulate employees’ curiosity, enabling them to generate novel and useful ideas and to address practical work-related problems more effectively.

Third, since performance feedback has been identified as a key boundary condition that amplifies the effect of mindfulness on employees’ curiosity and subsequent creativity, we recommend that leaders provide regular, targeted performance feedback. Effective feedback can help employees—particularly those with higher levels of mindfulness—quickly clarify critical work goals, identify gaps between current and desired knowledge, and determine areas for improvement (Gonzalez-Mulé et al., 2016), thereby guiding more focused exploration and learning. In this way, organizations are more likely to stimulate employees’ curiosity and enhance their creativity.

### 5.4 Limitations and future research directions

This study makes important theoretical and practical contributions, yet several limitations warrant consideration, which provide directions for future research. First, although we employed a time-lagged research design, we were unable to establish causal relationships among mindfulness, curiosity, and employee creativity. In addition, mindfulness and performance feedback were measured at the same time point, which limits our ability to fully establish temporal causality. Future research could employ longitudinal data or experimental designs to more clearly test causal relationships. Second, although we used a workplace mindfulness scale to measure employees’ mindfulness (Hülsheger and Alberts, 2021), which fits the research context, this scale mainly focuses on task-related situations. Future research could adopt or develop measures that better capture a broader range of workplace activities (e.g., including interpersonal and emotional aspects) to examine whether our findings can be replicated. Third, while this study identified workplace curiosity as an autonomous motivational mechanism linking mindfulness to creativity and ruled out the potential influence of intrinsic motivation in the model, other potential mechanisms (e.g., cognitive and emotional processes) were not examined. On the one hand, future research could extend our understanding by exploring affective pathways through which mindfulness influences creativity, including both positive and negative emotional processes. On the other hand, scholars can also examine cognitive mechanisms to provide additional insights into how mindfulness facilitates creativity, for instance, creative self-efficacy is often considered a more proximal driver of creativity (Gong et al., 2009). Fourth, although our findings highlight performance feedback as an important contextual factor, other boundary conditions may also play a role. In particular, leadership is commonly viewed as a key contextual factor that shapes and amplifies employees’ motivation and behavioral responses. Future research could further investigate the potential role of leadership in influencing the mindfulness–curiosity relationship. Finally, our sample was drawn from China, a cultural context characterized by relatively high levels of power distance, which may influence employees’ perceptions of and responses to performance feedback. Future research should test our model in other cultural settings to enhance the generalizability of our findings.

## Data Availability

The raw data supporting the conclusions of this article will be made available by the authors, without undue reservation.
